# Correlation between hepatitis B surface antibody (anti-HBs) in maternal blood and cord blood in newborn: a study on transplacental acquired maternal antibody

**DOI:** 10.1186/s12887-025-05569-w

**Published:** 2025-03-18

**Authors:** Yudith Setiati Ermaya, Eka Surya Nugraha, Dolvy Girawan, Nelly Amalia Risan, Muhammad Begawan Bestari, Raden Tina Dewi Judistiani, Tetty Yuniati, Dwi Prasetyo

**Affiliations:** 1 Department of Child Health, Faculty of Medicine, Padjadjaran University, Dr. Hasan Sadikin General Hospital, Bandung, West Java Indonesia; 2 Department of Internal Medicine, Faculty of Medicine, Padjadjaran University, Dr. Hasan Sadikin General Hospital, Bandung, West Java Indonesia; 3 Department of Public Health, Faculty of Medicine, Padjadjaran University, Bandung, West Java Indonesia

**Keywords:** Anti-HBs, Hepatitis B virus, Cord blood, Transplacental acquired maternal antibody

## Abstract

**Background:**

Hepatitis B Virus infection is a global health problem. Transplacental maternal antibodies can protect the infant early in life from infection. Objectives: This study investigates the correlation between maternal and infant Hepatitis B surface antibodies (anti-HBs) in pairs.

**Methods:**

This cross-sectional study measured anti-HBs in paired mother-infant samples. Blood samples were taken from the mother 3 h before delivery and from the newborn immediately after birth by cord blood, and they were then examined for anti-HBs using the Chemiluminescent Microparticle Immunoassay.

**Results:**

Transplacental transfer of maternal anti-HBs was analyzed in 79 mother-infant pairs. Seventeen mothers (21.5%) had positive anti-HBs and all cord blood of newborns from these mothers had anti-HBs detected. Overall, there were 44 (55.7%) newborn blood cords that were positive for anti-HBs. The geometric mean of anti-HBs cord blood titers in newborns with maternal anti-HBs titers < 10, ≥10, ≥ 100, and ≥ 1,000 mIU/mL were 52.42, 193.83, 437.12, and ≥ 1,000 mIU /mL respectively. This study showed a significant correlation in anti-HBs between mother and infant cord blood (*r* = 0.863; *p* < 0,001).

**Conclusions:**

Anti-HB antibodies measured in mother and infant cord blood were strongly correlated, demonstrating efficient transplacental antibody transfer to protect infants against Hepatitis B infection. Hepatitis B vaccination is required for mothers to obtain immunogenicity and babies to receive hepatitis B vaccination on time.

## Introduction

Hepatitis B Virus (HBV) is a viral infection that attacks the liver and is still a health problem it causes the death of 1.4 million people with hepatitis B infection through acute infection, cirrhosis and liver cancer every year [1]. Among all viral hepatitis, HBV infection is the most common with different disease severity levels and detected in bodily fluid where serum has the greatest HBV concentration [1, 2].

The transmission of HBV from an infected mother to her infant often occurs during the perinatal period in highly endemic areas [1]. In Asia, mother to child transmission (MTCM) is one of the main transmission routes contributing to majority of new chronic HBV carriers [4]. Perinatal exposure and MTCM of HBV have been shown to be the leading cause of new infections worldwide [5–7].

Hepatitis B surface antibody (HBsAb or Anti-HBs) if detected “positive” or “reactive” indicates that a person is protected against the hepatitis B virus. This protection can be the result of receiving the hepatitis B vaccine or natural infection, successfully recovering from a past hepatitis B infection [3, 8]. In several previous studies, no response or non-reactive, protective response, adequate response and high response to vaccines were regarded as anti-HBs titers < 10 international units per milliliter (mIU/mL), anti-HBs ≥ 10 mIU/mL, anti-HBs ≥ 100 mIU/mL and anti-HBs ≥ 1000 mIU/mL, respectively. Immunity to HBV infection is obtained when antibodies produced surpass a serological threshold of 10 mIU/mL [9, 10]. Antibodies (anti-HBs) can cross the placental barrier easily and are found in the umbilical cord blood and in the amniotic fluid. Given the efficient transplacental transfer and higher level of anti-HBs in infants than in mothers, it is reasonable that infants from mothers with anti-HBs positive are immune to HBV infection early in life [11], it is possible that IgG antibodies cross the placenta through a process mediated by activated neonatal Fc receptors (FcRn) during pregnancy. Active transplacental transfer begins early in pregnancy, and fetal IgG levels increase with advancing gestational age, becoming relatively low between 17 and 22 weeks, and eventually exceeding maternal plasma IgG levels at birth [12]. This study aimed to determine This study investigates the correlation is between maternal and infant Hepatitis B surface antibodies (anti-HBs) in pairs.

## Materials and methods

### Study design and population

This cross-sectional community study was conducted in Bandung City, West Java from May to September 2022, on pregnant women and their infant after birth as mother-baby pairs. Hepatitis B vaccination is carried out routinely in West Java. In this study, data on the characteristics of the subjects were collected using a questionnaire that was distributed to the mothers. The inclusion criteria were mothers whose Hepatitis B surface antigen (HBsAg) was negative. Blood samples from the mother were taken before delivery and from the newborns immediately after birth via the umbilical artery to collect umbilical cord blood with an average time interval of 6 h between paired samples.

The blood samples were then analyzed in the laboratory to identify the presence of the anti-HBs in mother-infant pairs. Anti-HBs examination was carried out using the Chemiluminescent Microparticle Immunoassay. The analysis data is based on percentage for total pairs and geometric mean titers (GMT). A positive status for anti-HBs level was defined as the presence of $\:\ge\:1$0 mIU/mL of this antibody. They were assessed as non-reactive if the Anti-HBs level <10 mIU/mL.

Written informed consent was obtained from each mother before delivery as the participants of the study. The study was approved by the Health Research Ethical Committee of Universitas Padjadjaran, IRB no.470/UN6.KEP/EC/2022.

### Statistical analysis

The statistical tests to describe relationships of the characteristics were Pearson Chi-square test, Fisher’s exact test, and Spearman’s rank for correlation coefficient. Statistical analysis used Epi Info v.3.5.4 with a *p*-value of < 0.05 considered significant.

## Results

Transplacental transfer of maternal anti-HBs was analyzed in 79 pairs of mother and infant. The range of maternal age in this study was 16–43 years old, the mean age of mother was 27.6 years. All babies were born spontaneously in a healthy condition with a birthweight ranged between 2,000 and 3,900 g. Of all mothers participating in this study, 17 (21.5%) were positive for anti-HBs and transferred anti-HBs to their babies, which was reflected in the rate of infants with anti-HBs positive in the cord blood of 44 (55.7%).

**Fig. 1:**
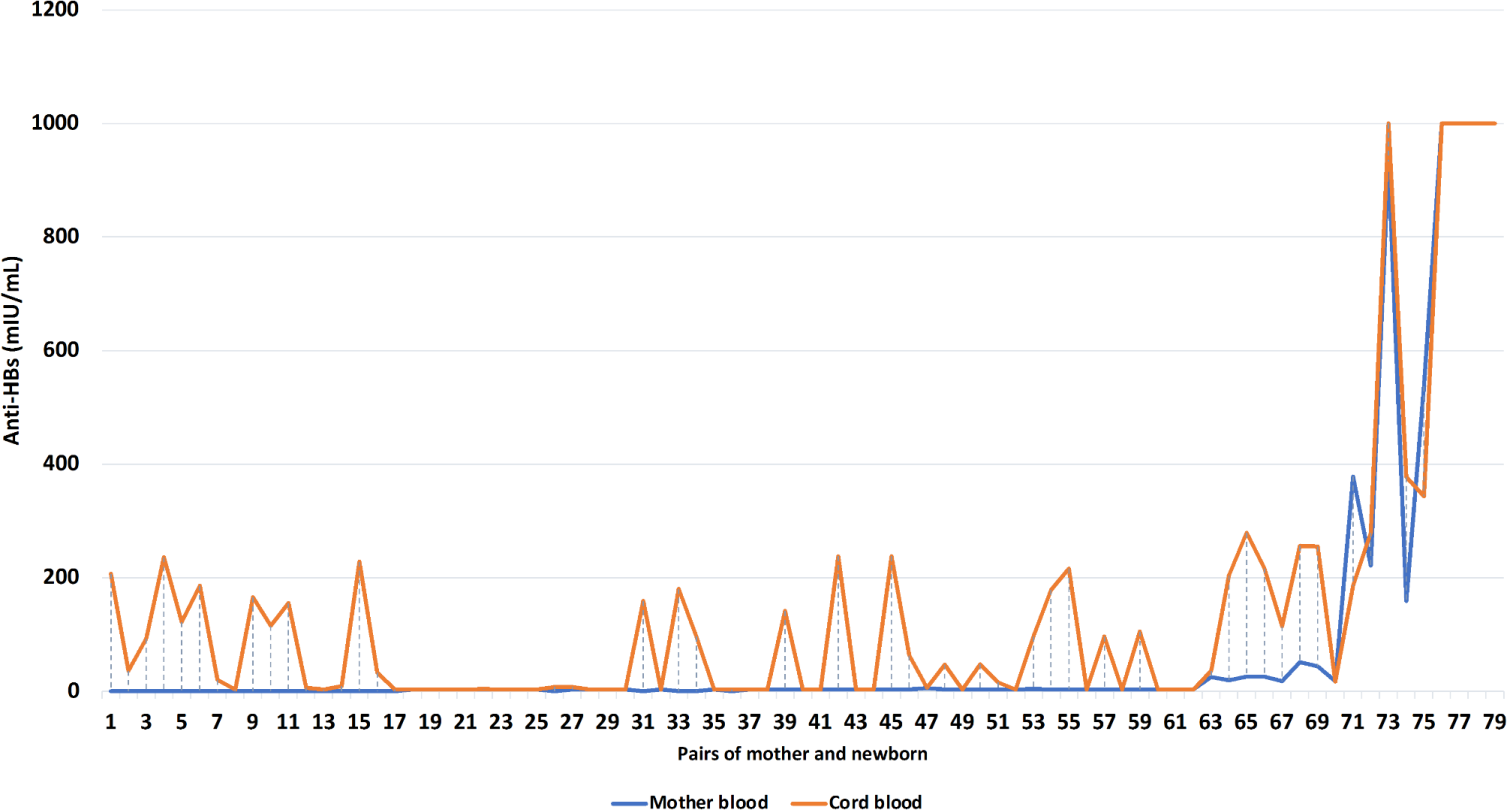
. The mother’s and the newborn’s cord blood anti-HBs titers in pair

The most common age distribution of mothers at birth was between 20 and 35 years. In this age group, 44 (55.7%) were positive for anti-HBs in the cord blood. The majority of the mothers delivered at the gestational age of ≥ 38 weeks to a term infant (*n* = 72, 91.1%) with most of them gave birth to their first-born child (*n* = 35, 45.6%). In terms of the education level, more than half of these women were junior high school graduates (*n* = 46, 58.2%) with a family income that was less than the minimum wage (*n* = 60, 75.9%). Most infants were born with a birthweight of more than 2,500 gr (*n* = 76, 96.2%). The maternal anti-HBs level during pregnancy and delivery ranged from 0.10 to > 1,000 mIU/mL, the mean level of anti-HBs is recorded at 82.82 mIU/mL. Meanwhile, the level in the infant cord blood ranged from 3.10 to > 1,000 mIU/mL, with an average Anti-HBs level of 141.96 mIU/mL. When further analysed, age at delivery, gestational age, maternal education level, family income, and birth weight were not different between infants with positive anti-HBs and negative anti-HBs cord blood (*p* > 0.05) (Table 1). The mother’s and the newborn’s cord blood anti-HBs titers are displayed in pairs (Fig. 1).

**Table 1:** Correlation between maternal characteristics and cord blood transplacental transferred maternal antibody

Characteristic	Total Pair *n* (%)	Anti-HBs in Cord Blood (mIU/mL)		*p*-value
		≥ 10 Positive	< 10 Negative	
Mother	79 (100)	44 (55.7)	35 (44.3)	
Age at delivery (year)				0.528*
< 20	5 (6.3)	4 (80.0)	1 (20.0)	
20–35	63 (79.7)	34 (54.0)	29 (46.0)	
> 35	11 (13.9)	6 (54.5)	5 (45.5)	
Mean (SD)	27.6 (6.5)	27.9 (6.4)	27.2 (6.7)	
Range	16–43	17–42	16–43	
Gestational age (week)				0.125**
<38	7 (8.9)	6 (85.7)	1 (14.3)	
≥ 38	72 (91.1)	38 (52.8)	34 (47.2)	
Mean (SD)	38.6 (1.5)	38.4 (1.7)	38.9 (1.1)	
Range	31–42	31–42	37–42	
Gravidity				0.316*
1	36 (45.6)	18 (50.0)	18 (50.0)	
2	16(20.2)	8 (50.0)	8 (50.0)	
3	18 (22.8)	13 (72.20)	5 (27.8)	
4	7 (8.9)	3 (42.9)	4 (57.1)	
6	2 (2.5)	2 (100)	0 (0)	
Education				0.483*
≤Elementary School	15 (19.0)	8 (53.3)	7 (46.7)	
Junior High School	46 (58.2)	28 (60.9)	18 (39.1)	
≥High School	18 (22.8)	8 (44.4.)	10 (55.6)	
Family income wage (Indonesian Rupiahs)				0.825*
< 3,623,778	60 (75.9)	33 (55.0)	27 (45.0)	
≥ 3,623,778	19 (24.1)	11 (57.9)	8 (42.1)	
Anti-HBs mother (mIU/mL)				
Positive	17 (21.5)			
Negative	62 (78.5)			
Geometric mean titer	82.82			
Range	0.10 - >1,000			
Cord blood (Baby)				
Birth weight (grams)				1.0**
< 2,500	3 (3.8)	2 (66.7)	1 (33.3)	
≥2,500	76 (96,2)	42 (55.3)	34 (44.7)	
Mean (SD)	3,103 (404.1)	3,060 ( 404.1)	3,157 (403.3)	
Range	2,000–3,900	2,300-3,800	2,000–3,900	
Anti-HBs blood cord (mIU/mL)				
Positive	44 (55.7)			
Negative	35 (44.3)			
Geometric mean titer	141.96			
Range	3.10 - >1,000			

*Person Chi-square test, **Fisher exact test

**Table 2:** Correlation between maternal Anti-HBs and newborn cord blood Anti-HBs

Maternal Anti-HBs	Total Pairs n (%) 79 (100)	Cord Blood Anti-HBs		*p*-value
		Positive ≥ 10 mIU/mL *n* = 44 (55.7)	Negative < 10 mIU/mL *n* = 35 (44.3)	
Positive 10 mIU/mL	17 (21.5)	17 (100.0)	0 (0.0)	< 0.001*
Negative < 10 mIU/mL	62 (78.5)	27 (43.5)	35 (56.5)	

*Persons Chi-square test

Table 2 shows that 17 (21.5%) mothers were positive for anti-HBs with all infants from these mothers (100%) also positive for anti-HBs. Meanwhile, among mothers who were anti-HBs negative 62 (78.5%), at 27 newborn were found to have cord blood that was positive for anti-HBs (43.5%) while the remaining 35 (56.5%) were anti-HBs negative. Results from Table 2. showed a significant correlation between maternal anti-HBs and newborn cord blood (*p* < 0.001).

**Table 3:** Anti-HBs level and seroprotection for hepatitis B in mother vs. Newborn cord blood

Variables	Total *n* = 79 100 (%)	Anti-HBs Range mIU/mL	Anti-HBs GMT mIU/mL	Seroprotection Average GMT mIU/mL		
**Mother (GMT mIU/mL)**						
non-reactive (< 10) No response	62 (78.5%)	< 0.10$\:-$5.67	2.09	21.5% 337.51		
low-reactive (≥10) Protective response	8 (7.6%)	17.89$\:-$51.26	36.45			
high-reactive (≥100) Adequate response	5 (6.3% )	159.01$\:-$933.42	474.87			
highly reactive (≥1000) High response	4 (5.1%)	> 1000	1000			
**Cord blood (GMT mIU/mL)**						
non-reactive (< 10) No response	35 (44.3% )	< 3.1$\:-$9.45	3.77	55.7% 246.49		
low-reactive (≥10) Protective response	12 (15.2% )	16.06$\:-$96.43	53.67			
high-reactive (≥100) Adequate response	27 (34.2%)	104.94$\:-$377.27	205.93			
highly reactive (≥1000) High response	5 (6.3%)	>1000	1000			

GMT: geometric mean titer

Although there were more non-reactive mother (78.5%), whereas in newborns it was found that 44.3% had antibodies below the protective threshold or did not have protection against HBV. This study found that mothers and babies have seroprotection (21.5 vs. 55.7%), with GMT (337.51 vs. 246.49) mIU/mL, it shows that there are transplacental antibodies from the mother which can increase immunity in the baby. This is very important in screening newborns to find out to what extent the baby has protection against hepatitis B infection. (Table 3). There is a correlation based on immunogenicity between the mother and newborn cord blood, *p* < 0,001) (Table 4).

**Table 4:** Correlation between maternal antibody immune response and cord blood

Maternal anti-HBs (mIU/mL)	Cord blood anti-HBs (mIU/mL) %					*p*-value
	≥ 1,000	101–999	≥ 10–100	< 10	Total	
≥ 1,000	4 (100.0)	N/A	N/A	N/A	4 (5.1)	< 0.001
101–999	1 (20.0)	4 (80.0)	N/A	N/A	5 (6.3)	
≥ 10–100	N/A	6 (75.0)	2 (25.0)	N/A	8 (10.1)	
< 10	N/A	17 (27.4)	10 (16.1)	35 (56.6)	62 (78.5)	
Total	5 (6.3)	27 (34.2)	12 (15.2)	35 (44.3)	79 (100.0)	

^*^Pearson Chi-Square. N/A: Not available

**Fig. 2:**
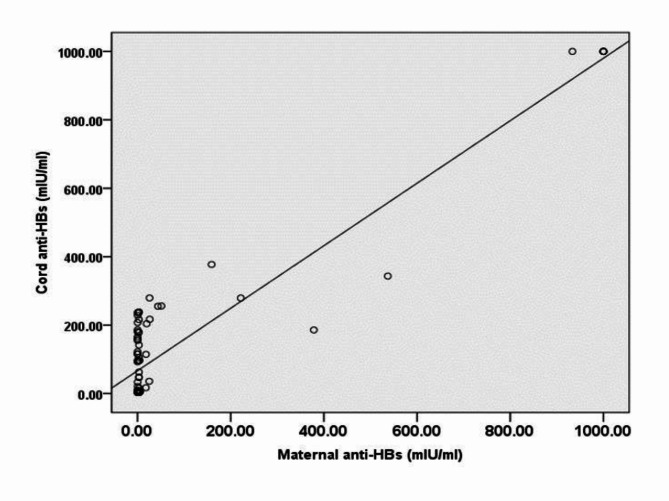
Maternal transplacental antibody (anti-HBs) and cord blood correlation. Linear regression analysis: Cord anti-HBs = 66.801 + 0.913*Maternal anti HBs (*r* = 0.863; *p* < 0,001, *n* = 79)

Figure 2 demonstrates a correlation between anti-HBs in maternal blood and infant cord blood, presenting the transplacental acquired antibodies. The anti-HBs in infant cord blood was found to be higher than that of the maternal blood. Anti-HBs level in infants born from mothers with level of $\:\ge\:1$0$\:-$100 mIU/mL had a geometric mean titer (GMT) of 193.83 mIU/mL (range: 17.06$\:-$279.37). This was similar to the GMT of the infant cord blood from mothers with the level of anti-HBs 101$\:-$999 mIU/mL (GMT = 437.11 mIU/mL; range: 185.93$\:-$1,000). All newborns delivered by mothers with an anti-HBs level of ≥ 1,000 mIU/mL (GMT = 1,000 mIU/mL) exhibit Anti-HBs in their cord blood. while those who were born from mothers with the level of anti-HBs <10 mIU/mL (GMT = 58.89 mIU/mL) demonstrated a mixture of anti-HBs negative and positive in their newborn cord blood (range: <0.31$\:-$9.45 vs. 16.06$\:-$237.81).

## Discussion

Anti-HBs antibody is developed after a natural infection of HBV or after HB vaccination that activates the human immune system through the HBsAg. Individuals who are anti-HBs positive are immunologically competent against the HBV infection [9]. In previous studies, 99.8% of maternal anti-HBs antibodies were transferred transplacental and passively passed on to their babies [13].

The maternal age range in this study is similar to a study in China. That gestational age was predominantly ≥ 38 weeks, just as in the previous study [11], Most of the participants were multiple gravidities, this is in line with research in Tanzania [14], the majority of junior high school graduates with low economic income status, this characteristic is similar to research in Brazil [15].

Of 79 mother-infant pairs, 17 mothers were found to have positive anti-HBs (21.5%) with all babies from these mothers were also positive for anti-HBs, it means that mothers with positive anti-HBs have babies who are born positive for anti-HBs. Newborns from mothers with an anti-HBs titer of $\:\ge\:\text{1,000}$ mIU/mL showed the highest GMT for anti-HBs. This association between maternal anti-HBs status and infant cord blood anti-HBs was significant with *p* <0.001. This is similar to the finding in a previous study in Athen that demonstrates the placental barrier can be easily crossed by anti-HBs [16].

Among mothers who were anti-HBs negative antibody levels < 10 mIU/mL (*n* = 62, 78.5%), given birth 27 infants with anti-HBs positive (43.5%), the study show 17 infants with antibody levels between 101 and 999 mIU/mL and 10 infants with antibody levels 10–100 mIU/mL, there are while the remaining 35 delivered to infants with anti-HBs negative (56.5%). This is similar to other studies that the anti-HBs in most babies is higher than that of their mothers, even though from previous examinations it was clear that the mother did not have hepatitis B infection as proven by a Hepatitis B examination and had not received hepatitis B vaccination, this is a very interesting thing to do further research. The presence of effective transplacental transfer and anti HBs level in Infant blood cord higher suggest that infants from mothers who are positive for anti-HBs have immunity and protection against HBV Infection in the first period of life [11, 16, 17]. The level of anti-HBs in the bloodstream is influenced by several factors that need to be recognized, especially in individuals with anti-HBs negative as well as in those with detectable but low levels of protective immunity [9].

In this study, meanwhile, there were also infants with an anti-HBs level of < 10 $\:\text{m}\text{I}\text{U}/\text{m}\text{L}$ in the cord blood (*n* = 35, 44.3%). The total number of mothers with Anti-HBs level of$\:\:\ge\:\text{1,000}\:\:\text{m}\text{I}\text{U}/\text{m}\text{L}$ during pregnancy and delivery was 4 (5.1%). This shows that with the presence of a high level of Anti-HBs, the mother can protect and provide immunity to her baby against Hepatitis B infection. This supports similar findings from a previous study [11].

Another interesting finding is that infants born to mothers with anti-HBs level of $\:\ge\:1$0$\:-$100 mIU/mL had anti-HBs GMT 193.83 mIU/mL (range: 17.06$\:-$279.37). This was similar to the GMT of the infant cord blood from mothers with anti- HBs level of 101-999 mIU/mL (GMT = 437.12 mIU/mL; range: 185.93–1,000). All infants from mothers with an anti-HBs level of $\:\ge\:$1,000 mIU/mL (GMT = 1,000 mIU/mL) have anti-HBs in their cord blood while those who were born from mothers with an anti-HBs level of <10 mIU/mL (GMT = 52.42 mIU/mL) demonstrated a mixed of anti-HBs negative and positive in their infant cord blood (range: <0.31$\:-$9.45 vs. 16.06 $\:-$>1,000). This is in line with previous research in Brazil [15].

This study showed the presence of a higher level of anti-HBs in the cord blood (55.7%) indicating the presence of transplacental acquired maternal antibody, with Seroprotection for Hepatitis B in mother vs. newborn cord blood (21.5% vs. 55.7%). In a previous study, a passive transfer of anti-HBs from mother to infant occurs in 59% of newborns, however, this antibody disappears rapidly in infants down to only 23% of these infants still have detectable antibodies after 3 months [18] and all of them (100%) in 8th month became negative [13].

Findings of this study emphasize the importance of maternal immunity against HBV early in life. However, with the waning protection of this immunity with time, it is very important to complete hepatitis B immunization in infancy to obtain immunity against hepatitis B up to adulthood.

## Conclusions

In conclusions, there was a correlation between maternal anti-HBs and infant cord blood anti-HBs, this study shows that anti-HBs levels in newborns cord blood are higher than in mothers, which indicates the presence of maternal antibodies obtained transplacental which are transferred actively, meaning that babies from mothers who are anti-HBs positive are immune to HBV infection early in life. Hepatitis B vaccination is required for mothers to obtain immunogenicity and for babies to get hepatitis B vaccination promptly on schedule.

## Data Availability

No datasets were generated or analysed during the current study.

## Abbreviations

Anti HBs: Hepatitis B surface antibodies
HBsAg: Hepatitis B surface antigen
GMT: Geometric mean titers
HBV: Hepatitis B virus
MTCM: Mother to child transmission
